# An increase in erythromycin resistance in methicillin-susceptible *Staphylococcus aureus* from blood correlates with the use of macrolide/lincosamide/streptogramin antibiotics. EARS-Net Spain (2004–2020)

**DOI:** 10.3389/fmicb.2023.1220286

**Published:** 2023-09-26

**Authors:** Achraf El Mammery, Eva Ramírez de Arellano, Javier E. Cañada-García, Emilia Cercenado, Laura Villar-Gómara, Verónica Casquero-García, Silvia García-Cobos, José Antonio Lepe, Enrique Ruiz de Gopegui Bordes, Jorge Calvo-Montes, Nieves Larrosa Escartín, Rafael Cantón, María Pérez-Vázquez, Belén Aracil, Jesús Oteo-Iglesias

**Affiliations:** ^1^Laboratorio de Referencia e Investigación en Resistencia a Antibióticos e Infecciones Relacionadas con la Asistencia Sanitaria, Centro Nacional de Microbiología, Instituto de Salud Carlos III, Madrid, Spain; ^2^Escuela Internacional de Doctorado, Ciencias Biomédicas y Salud Pública - IMIENS (UNED), Madrid, Spain; ^3^CIBER de Enfermedades Infecciosas (CIBERINFEC), Instituto Salud Carlos III, Madrid, Spain; ^4^Servicio de Microbiología, Hospital General Universitario Gregorio Marañón, Madrid, Spain; ^5^CIBER de Enfermedades Respiratorias (CIBERES), Instituto de Salud Carlos III, Madrid, Spain; ^6^Agencia Española de Medicamentos y Productos Sanitarios (AEMPS), Madrid, Spain; ^7^Servicio de Microbiología, Hospital Universitario Virgen del Rocío, Sevilla, Spain; ^8^Servicio de Microbiología, Hospital Universitario Son Espases, Palma de Mallorca, Spain; ^9^Servicio de Microbiología, Hospital Universitario Marqués de Valdecilla-IDIVAL, Santander, Spain; ^10^Servicio de Microbiología, Hospital Universitario Vall d’Hebron, Barcelona, Spain; ^11^Servicio de Microbiología, Hospital Universitario Ramón y Cajal, Madrid, Spain

**Keywords:** *Staphylococcus aureus*, macrolides, ST398, *ermT*, EARS-Net, antibiotic resistance, antibiotic consumption

## Abstract

**Objectives:**

To describe and analyse erythromycin resistance trends in blood isolates of *Staphylococcus aureus* (EARS-Net Spain, 2004–2020) and the association of these trends with the consumption of macrolide, lincosamide, and streptogramin B (MLS_B_) antibiotics. To assess molecular changes that could be involved in erythromycin resistance trends by whole genome analysis of representative isolates.

**Materials and methods:**

We collected antibiotic susceptibility data for all first-blood *S. aureus* isolates in patients from 47 Spanish hospitals according to EARS-Net criteria. MLS_B_ antibiotic consumption was obtained from the Spanish Agency for Medicines and Medical Devices (2008–2020). We sequenced 137 representative isolates for core genome multilocus sequence typing, resistome and virulome analysis.

**Results:**

For the 36,612 invasive *S. aureus* isolates, methicillin resistance decreased from 26.4% in 2004 to 22.4% in 2020. Erythromycin resistance in methicillin-susceptible *S. aureus* (MSSA) increased from 13.6% in 2004 to 28.9% in 2020 (*p* < 0.001); however, it decreased from 68.7 to 61.8% (*p* < 0.0001) in methicillin-resistant *S. aureus* (MRSA). Total consumption of MLS_B_ antibiotics increased from 2.72 defined daily doses per 1,000 inhabitants per day (DID) in 2014 to 3.24 DID in 2016. By WGS, the macrolide resistance genes detected were *erm* (59.8%), *msrA* (46%), and *mphC* (45.2%). The *erm* genes were more prevalent in MSSA (44/57, 77.2%) than in MRSA (38/80, 47.5%). Most of the *erm* genes identified in MSSA after 2013 differed from the predominant *ermC* gene (17/22, 77.3%), largely because *ermT* was significantly associated with MSSA after 2013 (11/29, 37.9%). All 13 *ermT* isolates in this study, except one, belonged to ST398 and came from 10 hospitals and six Spanish provinces.

**Conclusion:**

The significant increase in erythromycin resistance in blood MSSA correlated with the consumption of the MLS_B_ antibiotics in Spain. These preliminary data seem support the hypothesis that the human ST398 MSSA clade with *ermT*-mediated resistance to erythromycin may be involved in this trend.

## Introduction

The ever-increasing prevalence of antibiotic resistance in bacteria is a serious concern that requires an international approach to management. Hence, the World Health Organization (WHO) and the European Commission both recognize the importance of understanding the emergence and determinants of resistance and the need for control strategies. In Europe, the European Antimicrobial Resistance Surveillance Network (EARS-Net) has collected antimicrobial susceptibility data for isolates from routine blood and cerebrospinal fluid cultures since 1988 (Gagliotti et al., 2011). Funded by the European Commission, EARS-Net is a European network of national surveillance systems coordinated by the European Centre for Disease Prevention and Control (ECDC), whose goal is to collect comparable, reliable data to identify variations in antimicrobial resistance over time and space, providing a basis for infection prevention and control programs (ECDC, 2022).

An EARS-Net indicator organism is *Staphylococcus aureus*, which is a pathogen of major clinical importance for nosocomial infections. Since the introduction of antibiotics in clinical practice, *S. aureus* has progressively developed resistance to the most frequently used antibiotics. It is noteworthy that the first clinical isolate of methicillin-resistant *S. aureus* (MRSA) was reported in 1961, just 1 year after the launch of methicillin (Jevons, 1961). WHO considers MRSA the most important Gram-positive bacterial strain (Priority 2/High) for research and the development of new antibiotics (Tacconelli et al., 2018).

Macrolides, lincosamides, and streptogramin B (MLS_B_) are alternative antibiotics used to treat severe staphylococcal infections, mainly in penicillin-allergic patients, along with vancomycin and the combination of linezolid and rifampicin (Ji and Xu, 2021). Macrolide resistance emerges quickly and persists, even after a short course of therapy (Malhotra-Kumar et al., 2009; Van Heirstraeten et al., 2012). Total MLS_B_ consumption did not change significantly in Europe from 2007 to 2017 (Adriaenssens et al., 2021); however, the consumption of long-acting macrolides increased, and seasonal variation was high, suggesting that MLS_B_ antibiotics were prescribed inappropriately in many countries (Adriaenssens et al., 2021).

Staphylococcal resistance to macrolides includes target-site modification by methylation, such as erythromycin ribosome methylase (*erm*) genes; antibiotic efflux pumps, such as ABC-F proteins (*msr* genes) and major facilitator superfamily transporters (*mef* genes); and drug inactivation, such as phosphotransferases (*mph* genes) and esterases (*ere* genes) (Feßler et al., 2018; Lade et al., 2022). The *erm* gene products methylate specific targets in the 23S rRNA, preventing the antibiotic from binding to its ribosomal target (di Bonaventura et al., 2019). This is the most widespread mechanism of resistance to macrolides, lincosamides and streptogramin B cross-resistance (MLS_B_ phenotype). The most common *erm* genes in *S. aureus* are *erm*(C) and *erm*(A), which can be either constitutive or inducible (Fyfe et al., 2016; di Bonaventura et al., 2019).

The goals of this study were (i) to describe and analyse erythromycin resistance trends in the blood isolates of *S. aureus* collected by EARS-Net Spain (2004–2020), (ii) to compare erythromycin resistance with trends in the consumption of the MLS_B_ antibiotic family (J01F family), and (iii) to characterize antibiotic resistance genes and the prevalence of resistant clones by whole genome sequencing (WGS) in a representative sample of *S. aureus* isolates.

## Materials and methods

### Antibiotic resistance

Forty-seven Spanish EARS-Net hospitals collected antibiotic susceptibility data for all *S. aureus* isolated from the first blood collected from each patient from 2004 to 2020. Using EARS-Net criteria, we selected hospitals that were distributed evenly across the country (TESSy-The European Surveillance System, 2022). These hospitals serve ~13.5 million people, ~32% of the total Spanish population, and are representative of 15 of the 17 Spanish autonomous communities. Each laboratory identified the isolates and tested their susceptibilities according to standard microbiological procedures using commercial microdilution broth assays (Gagliotti et al., 2011, 2021). Results were interpreted according to EUCAST criteria.^1^ A quality assurance exercise (UK National External Quality Assessment Scheme) was performed annually to ensure comparable results among the hospital laboratories. All the *S. aureus* isolates included in EARS-Net have information on susceptibility to methicillin (the only mandatory indicator), but the number of isolates tested for other antibiotics may vary (Table 1).

**Table 1:** Evolution of antibiotic resistance of blood isolates of *Staphylococcus aureus* in according to EARS-Net (Spain, 2004–2020).

Antibiotics					
Year (*n*)	Methicillin*	Erythromycin*	Ciprofloxacin*	Clindamycin*	Gentamicin*
2004 (1,604)	26.4 (423/1,604)	31.2 (496/1,592)	27.7 (348/1,259)	13.4 (88/657)	8.5 (134/1,586)
2005 (1,388)	27.3 (379/1,388)	31.3 (429/1,371)	27.3 (265/973)	12.5 (124/998)	6.9 (89/1,292)
2006 (1,620)	25.0 (405/1,620)	27.6 (437/1,584)	27.0 (303/1,125)	11.0 (143/1,303)	6.7 (101/1,520)
2007 (1,730)	25.5 (441/1,730)	26.0 (446/1,718)	27.0 (355/1,317)	8.7 (130/1,495)	5.7 (94/1,648)
2008 (1,756)	26.6 (467/1,756)	29.6 (505/1,708)	30.6 (376/1,229)	12.2 (181/1,487)	6.9 (108/1,567)
2009 (1,835)	25.9 (475/1,835)	28.2 (511/1,815)	32.1 (380/1,184)	10.3 (153/1,491)	7.4 (128/1,740)
2010 (2,115)	25.2 (533/2,115)	28.3 (599/2,115)	30.9 (421/1,365)	12.0 (227/1,897)	6.1 (125/2,052)
2011 (2,147)	22.6 (485/2,147)	24.9 (528/2,121)	28.4 (429/1,513)	10.2 (216/2,115)	5.2 (110/2,123)
2012 (2,194)	25.0 (549/2,194)	27.2 (584/2,148)	29.2 (508/1,742)	8.2 (179/2,183)	5.2 (114/2,211)
2013 (2,113)	23.4 (519/2,113)	34.4 (658/1,913)	24.5 (428/1,751)	8.9 (177/1,990)	3.8 (81/2,113)
2014 (2,235)	23.2 (463/2,235)	35.4 (699/1,976)	25.1 (433/1,726)	8.7 (173/1,994)	3.8 (80/2,105)
2015 (2,290)	26.7 (561/2,290)	39.6 (886/2,238)	31.2 (557/1,786)	12.5 (263/2,110)	4.7 (107/2,283)
2016 (2,270)	25.4 (611/2,270)	35.9 (845/2,168)	27.8 (501/1,805)	11.4 (225/1,978)	3.3 (75/2,269)
2017 (2,206)	26.3 (580/2,206)	41.6 (787/1,894)	30.5 (485/1,593)	8.3 (145/1,753)	5.6 (108/1,939)
2018 (3,189)	23.5 (749/3,189)	39.9 (1,248/3,128)	26.8 (801/2,989)	15.0 (394/2,625)	4.2 (126/3,022)
2019 (2,847)	20.8 (592/2,847)	39.8 (1,074/2,699)	22.8 (486/2,135)	15.5 (325/2,098)	4.5 (104/2,315)
2020 (3,073)	22.4 (688/3,073)	36.1 (1,082/2,998)	26.1 (612/2,346)	18.4 (533/2,896)	5 (128/2,556)
Total (36,612)	24.4 (8,920/36,612)	32.7(11,814/35,186)	27.6 (7,688/27,838)	11.8 (3,676/31,070)	5.5 (1888/34,328)
*X*_2_ for trend (P)	23.20 (<0.0001)	292.5 (<0.0001)	10.45 (0.001)	78.43 (<0.0001)	66.34 (<0.0001)

*The resistance results to the different antibiotics tested are expressed as percentages; the number of resistant isolates/number of total isolates tested for that antibiotic is shown in parentheses.

### Antibiotic consumption

Community consumption of the MLS_B_ antibiotic family (WHO code J01F) according to the public health prescriptions for the period 2012–2020 was provided by the Spanish National Action Plan on Antimicrobial Resistance (PRAN), coordinated by the Spanish Agency for Medicines and Medical Devices (AEMPS) from the Ministry of Health, and was obtained from the database of retail pharmacy sales from National Health System prescriptions (covering near 100% of the Spanish population). In addition, the community use of MLS_B_ antibiotics from private health prescriptions was obtained from market research companies provided by the PRAN for the period 2012–2020. MLS_B_ hospital dispensing data were also available from public hospital pharmacies and market research companies for private hospital pharmacies, both provided by the PRAN.

The consumption data were tabulated, and the number of units was converted into defined daily doses (DDD) of active drug ingredients according to WHO methodology (WHO Collaborating Centre for Drug Statistics Methodology, 2023). The number of DDD per 1,000 inhabitants per day (DID) was calculated for each active drug ingredient.

### WGS of *Staphylococcus aureus* isolates

To study the population structure and macrolide resistance genes in *S. aureus* by WGS, we selected a total of 137 isolates sent to the *S. aureus* Reference Laboratory of the Spanish National Microbiology Centre according to the following characteristics: 46 MRSA isolated before 2013, 34 MRSA isolated after 2013, 28 MSSA isolated before 2013, and 29 MSSA isolates after 2013. These isolates were selected to have a broad geographic representation, and to represent MSSA and MRSA from periods before and after the start of the trend change in erythromycin resistance detected by EARS-Net at MSSA in 2013.

These isolates, which were isolated from blood (41, 29.9%), skin (35, 25.5%), the respiratory tract (34, 24.8%), and other samples (27, 19.7%), came from a total of 71 healthcare centers in 30 Spanish provinces.

### Genomic library preparation and sequence analysis

Genomic library preparation and sequence analysis were conducted as described (Pérez-Vázquez et al., 2019). Raw sequence data were submitted to the European Nucleotide Archive (PRJEB61102). The quality of the short reads was assessed using FASTQC, and they were assembled into contigs with Unicycler 0.4.8 (Wick et al., 2017). The quality of the assembly was assessed with QUAST.^2^ Prokka v1.14-beta (Seemann, 2014) was used for automatic *de novo* assembly annotation.

### Phylogenetic analyses

Sequence types (STs) were calculated according to the multilocus sequence typing (MLST) scheme of the *Public databases for molecular typing and microbial genome diversity* (PubMLST)^3^ using Ariba v2.6.2 (Hunt et al., 2017). A simple diversity index (SDI; Gastmeier et al., 2006) was applied to analyze population diversity. Core genome MLST (cgMLST), consisting of 1861 targets for *S. aureus* provided by SeqSphere+3.5.0 (Ridom, Münster, Germany), was performed with the 137 sequenced isolates. Additionally, ST398 isolates from this study were analyzed using cgMLST together with a collection of 239 *S. aureus* of this ST downloaded from the NCBI database using “chromosome” and “complete” as filtering criteria for assembly level and the absence of *mec* genes as the genotypic criterion.

### Analysis of antimicrobial resistance and virulence genes

Antibiotic resistance genes were analyzed by Ariba v2-6.2 using the CARD database^4^ and ResFinder (CGE server^5^). Virulence genes were analyzed with the previous methodology using the database Virulencefinder_db.^6^

### Statistical analysis

The significance of the trends in macrolide resistance was calculated by the χ^2^ test for trend. Trends in J01F antibiotic family consumption were examined by simple linear regression analysis. The strength of the association between MLS_B_ antibiotic use and erythromycin resistance was determined by linear regression analysis (Cuevas et al., 2011). The resistance proportion was transformed to the natural logarithm of the odds of resistance. The log of the odds of resistance (as the dependent variable) was expressed as a simple linear function of the independent variable (antibiotic use) (Cuevas et al., 2011). Macrolide resistance for 2013–2020 was correlated with antibiotic use in the prior year. Fisher’s exact test was used to compare the prevalence of resistance or virulence genes by groups. *p* values < 0.05 were considered statistically significant. Statistical analyses were performed using GraphPad Prism software v.7.02 (GraphPad Software Inc., San Diego, CA, United States).

## Results

### Sentinel hospitals, patients, and isolates

Most of the participating hospitals (29, 61.7%) were tertiary university hospitals with >500 beds, and 9 (19.1%) had >1,000 beds; however, three primary (6.4%) and 15 secondary (31.9%) hospitals also participated. Data on 36,612 consecutive *S. aureus* blood infections, corresponding to the same number of patients, were collected between 2004 and 2020; 23,887 patients (65.2%) were males, and 34,146 (93.3%) were adults (>14 years). The blood cultures were obtained from patients in internal medicine (45.9%), the emergency room (28.2%), intensive care units (8.7%), surgery (5.9%), pediatrics (3.1%), and other departments (8.2%).

### Antibiotic resistance trends in blood isolates of *Staphylococcus aureus*

Global resistance rates to methicillin, erythromycin, ciprofloxacin, gentamicin, and clindamycin were 24.4% (36,612 isolates analyzed), 32.7% (35,186 analyzed), 27.6% (27,838 analyzed), 11.8% (31,070 analyzed), and 5.5% (34,328 analyzed), respectively. Methicillin resistance decreased from 26.4% in 2004 to 22.4% in 2022 (Table 1) (*p* < 0.0001). There was a similar decrease in resistance to other antibiotics, including ciprofloxacin (from 27.7% in 2004 to 26.1% in 2020, *p* = 0.001) and gentamicin (from 8.5% in 2004 to 5.5% in 2020, *p* < 0.0001) (Table 1). For MLS_B_ antibiotics monitored in EARS-Net, erythromycin resistance increased from 31.2% in 2004 to 36.1% in 2020 (*p* < 0.0001; the highest value of the series was 41.6% in 2017), and clindamycin resistance increased from 13.4% in 2004 to 18.4% in 2020 (the highest value) (*p* < 0.0001) (Table 1).

**Figure 1:**
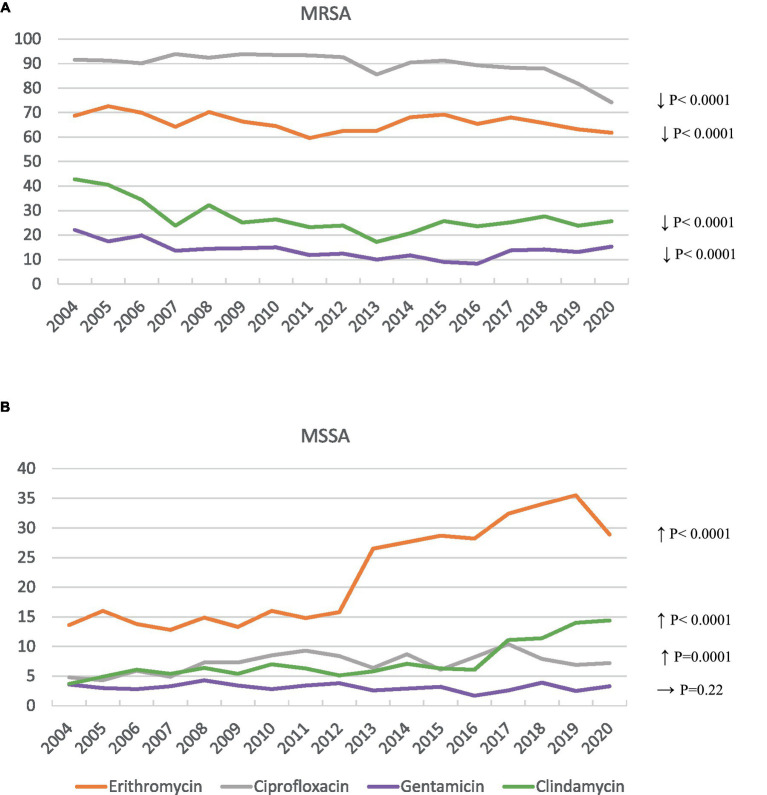
Antibiotic resistance trends in MRSA **(A)** and MSSA **(B)** blood isolates. **(A)** Shows significant decrease in resistance to ciprofloxacin, gentamicin, erythromycin and clindamycin; while **(B)** displays significant increase in resistance to erythromycin, clindamycin and ciprofloxacin.

There was greater resistance to ciprofloxacin, gentamicin, erythromycin, and clindamycin in MRSA isolates (89.5, 13.9, 66, and 27.1%, respectively) than in MSSA isolates (7.2, 3.1, 21.9, and 7.4%, respectively) (*p* < 0.001). However, between 2004 and 2020, MRSA isolates showed a significant decrease in ciprofloxacin resistance, from 91.6 to 74.2% (χ^2^ for trend = 76.7, *p* < 0.0001), in gentamicin resistance, from 22.1 to 15.3% (χ^2^ for trend = 21.9, *p* < 0.0001), in erythromycin resistance, from 68.7 to 61.8% (χ^2^ for trend = 25.7, *p* < 0.0001), and in clindamycin resistance from 42.8 to 25.6% (χ^2^ for trend = 26.79, *p* < 0.0001) (Figure 1). In contrast, MSSA isolates showed an increase in erythromycin resistance from 13.6% in 2004 to 28.9% in 2020 (χ^2^ for trend = 747.9, *p* < 0.0001), with the peak in 2019 at 35.5%. This trend was also seen for clindamycin resistance, from 3.7% in 2004 to 14.4% in 2020 (χ^2^ for trend = 208.9, *p* < 0.0001), and, although more moderate, for ciprofloxacin resistance, from 4.8% in 2004 to 7.2% in 2020 (χ^2^ for trend = 14.6; *p* = 0.0001). No significant variations over time in gentamicin resistance in MSSA isolates were identified (Figure 1).

### Macrolides, lincosamides, and streptogramins consumption

Community use of MLS_B_ antibiotics (WHO code J01F), including both public and private health prescriptions, increased in Spain from 2.61 DIDs in 2012 to 3.22 DIDs in 2016 (22.9%, *r*^2^ = 0.91, *p* = 0.012). It stabilized at around ~3.10 DIDs in 2017–2018 and decreased to 2.80 DIDs in 2019 prior to the COVID-19 pandemic in 2020, the year with the lowest use (2.04 DID) (Table 2). Nosocomial consumption of MLS_B_ between 2012 and 2020 (Table 2) increased from 0.11 DIDs (2012) to 0.14 DIDs (2019) (27.3%, *r*^2^ = 0.84, *p* = 0.006) with a peak (0.17 DIDs) in 2020 likely due the pandemic and the use of macrolides in admitted COVID-19 patients. The total consumption of MLS_B_ antibiotics (community and hospital consumption) increased between 2012 (2.72 DID) and 2016 (3.34 DID) (22.8%, *r*^2^ = 0.91, *p* = 0.01) stabilizing between 2017 and 2018 at ~3.23 DID (Table 2).

**Table 2:** Community and nosocomial consumption of macrolide, lincosamine, and streptogramin antibiotics group (WHO code J01F).

Year	Community consumption from public health prescriptions	Community use from health insurances prescriptions	Community consumption from private health prescriptions	Total community consumption	Nosocomial consumption	Total consumption
2012	1.72	0.17	0.72	2.61	0.11	2.72
2013	1.76	0.18	0.75	2.69	0.10	2.79
2014	1.86	0.18	0.78	2.82	0.10	2.92
2015	2.14	0.20	0.87	3.21	0.11	3.32
2016	2.14	0.19	0.89	3.22	0.12	3.34
2017	2.10	0.18	0.82	3.10	0.13	3.23
2018	2.10	0.18	0.80	3.08	0.14	3.22
2019	1.93	0.16	0.71	2.80	0.14	2.94
2020	1.42	0.10	0.52	2.04	0.17	2.21

Expressed in DDD per 1,000 inhabitants per day (DIDs).

### Correlation of erythromycin resistance in MSSA with total use of J01F antibiotics

**Figure 2:**
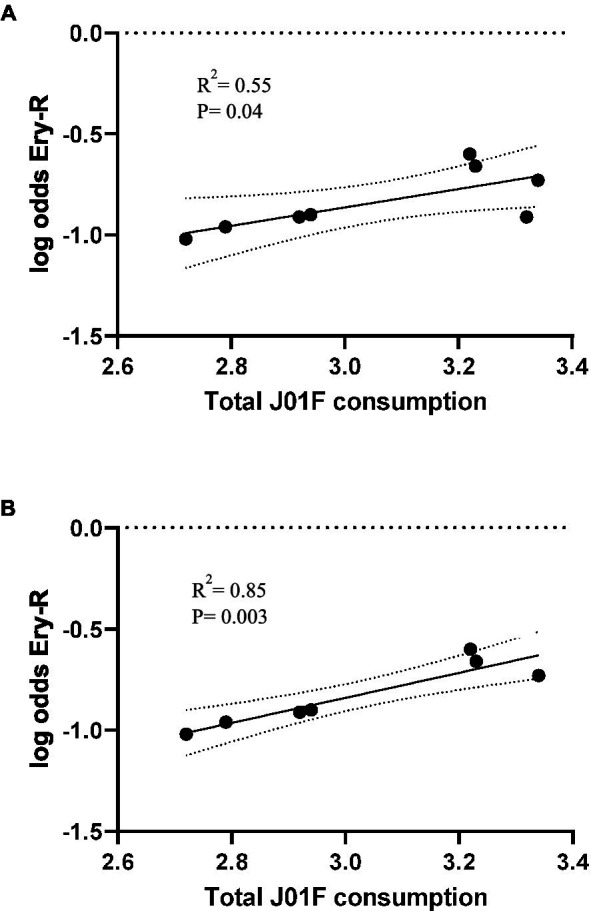
Occurrence of erythromycin resistance in *S. aureus* causing blood infections (years 2013–2020), plotted against total use of family J01F antibiotics (2012–2019) in Spain with 95% confidence intervals **(A)**. In **(B)**, the outlier erythromycin resistance data of 2016 (consumption of 2015) is excluded. Consumption is expressed in DIDs, DDDs/1000 inhabitants/day. Log odds is the natural logarithm of the OR.

The rates of erythromycin resistance in MSSA from 2013 to 2020 correlated with the total use of the J01F family of antibiotics (*r*^2^ = 0.55, *p* = 0.04) in the previous year (2012–2019) (Figure 2). However, when the resistance data for 2016 (representing atypical outlier data of unknown origin) were removed from the series, the correlation improved (*r*^2^ = 0.85, *p* = 0.003) (Figure 2).

### Resistance genes in erythromycin-resistant *Staphylococcus aureus* isolates by WGS

WGS of the 137 isolates identified *erm* genes in 83 (60.6%) isolates, a *msr*(A) gene in 63 (46%), and an *mph*(C) gene in 62 (45.2%). Five *erm* genes were identified, including *erm*(C) (49, 35.8%), *erm*(A) (21, 15.3%), *erm*(T) (13, 9.5%), *erm*(B) (one, 0.7%), and *erm*(X) (one, 0.7%); two isolates had two *erm* genes (Supplementary Table S1). A total of 61 isolates (44.5%) had both *msr*(A) and *mph*(C) genes, and 9 (6.6%) also carried an *erm* gene (Table 3). Of the 73 erythromycin-resistant isolates that had only *erm* genes, 19 (26%) showed a constitutive expression (84.2% had the *erm*(C) gene), and 54 (74%) were inducible (48.1% had the *erm*(C) gene). This predominance of constitutive *erm*(C) genes was statistically significant (*p* = 0.01). The *erm* genes were more prevalent in MSSA (45/57, 78.9%) than in MRSA (38/80, 47.5%) (*p* = 0.0007), whereas *msr*(A) predominated in MRSA (50/80, 62.5%) vs. MSSA (13/57, 22.8%) (*p* < 0.0001) (Table 3). There was no significant difference in macrolide resistance genes present in the isolates collected before or after 2013, although the *erm* genes were more prevalent after 2013 (41/63; 65.1%) than before 2013 (42/74, 56.7%).

**Table 3:** Distribution of macrolide and lincosamide resistance genes and main sequence types in *S. aureus* according to the groups studied.

Isolates (*n*)	*erm* genes (%)*	*msr* (A) gene (%)	*mph*(C) gene (%)	*cfr* gene (%)	*lnu* genes (%)	*vga* genes (%)	Total STs (SDI)	ST125 (%)	ST5 (%)	ST398 (%)	ST1 (%)	ST30 (%)	ST8
Total (137)	83 (60.6%) (49 *erm*(C))	63 (46%)	62 (45.2%)	2 (1.5%)	6 (4.4%)	1 (0.7%)	30 (21.9%)	45 (32.8%)	16 (11.7%)	14 (10.2%)	11 (8%)	8 (5.8%)	8 (5.8%)
MRSA (80)	38 (47.5%) (32 *erm*(C))	50 (62.5%)	50 (62.5%)	0	4 (5%)	1 (1.2%)	15 (18.7%)	40 (50%)	9 (5.6%)	3 (3.7%)	8 (10%)	0	7 (8.8%)
MSSA (57)	45 (78.9%) (17 *erm*(A))	13 (22.8%)	12 (21%)	2 (3.5%)	2 (3.5%)	0	21 (36.8%)	5 (8.8%)	7 (12.3%)	11 (19.3%)	3 (5.3%)	8 (14%)	1 (1.8%)
Before 2013 (74)	42 (56.7%) (27 *erm*(C))	38 (51.3%)	37 (50%)	0	3 (4.1%)	1 (1.4%)	23 (31%)	31 (41.9%)	7 (9.5%)	1 (1.3%)	3 (4%)	5 (6.8%)	7 (9.5%)
After 2013 (63)	41 (65.1%) (22 *erm*(C))	25 (39.7%)	25 (39.7%)	2 (3.2%)	3 (4.8%)	0	17 (27%)	14 (22.2%)	9 (14.3%)	13 (20.6%)	8 (12.7%)	3 (4.8%)	1 (1.6%)
MRSA before 2013 (46)	19 (41.3%) (16 *erm*(C))	33 (71.7%)	32 (69.6%)	0	2 (4.3%)	1 (2.2%)	11 (24%)	27 (58.7%)	3 (6.5%)	0	2 (4.3%)	0	7 (15.2%)
MSSA before 2013 (28)	23 (82.1%) (11 *erm*(A))	5 (17.9%)	5 (17.9%)	0	1 (3.6%)	0	16 (57.1%)	4 (14.3%)	4 (14.3%)	1 (3.6%)	1 (3.6%)	5 (17.9%)	0
MRSA after 2013 (34)	19 (55.9%) (16 *erm*(C))	17 (50%)	18 (52.9%)	0	2 (5.9%)	0	8 (23.5%)	13 (38.2%)	6 (17.6%)	3 (8.8%)	6 (17.6%)	0	0
MSSA after 2013 (29)	22 (75.9%) (11 *erm*(T))	8 (27.6%)	7 (24.1%)	2 (6.9%)	1 (3.4%)	0	13 (44.8%)	1 (3.4%)	3 (10.3%)	10 (34.5%)	2 (6.9%)	3 (10.3%)	1 (3.4%)

*The most frequent erm gene by group is detailed in brackets.

For the MSSA isolates collected after 2013, the group showing the most EARS-Net-based increase in erythromycin resistance, *erm* gene prevalence was 75.9% of (22/29), similar to MSSA strains isolated before 2013 (23/28, 82.1%) (Table 3). However, after 2013, the *erm* genes in MSSA were mostly different from the predominant *erm*(C) and included 11 strains with *erm*(T) and six with *erm*(A) (17/22, 77.3%) (Table 3; Supplementary Table S1). In fact, the *erm*(T) gene was significantly associated with MSSA isolated after 2013 (11/29, 37.9%), whereas it was identified in only two of the other isolates (2/108, 1.8%) (*p* < 0.0001). All 13 isolates with *erm*(T), except one, belonged to ST398 and were collected from 10 hospitals in six different Spanish provinces. The sequence of the genetic environment of *erm*(T) genes identified the *rep13* gene, which is involved in plasmid replication.

Nine isolates (6.6%) carried resistance genes for lincosamides (Supplementary Table S1), including lincosamide nucleotidyltransferases (*lnu*) genes in six isolates [five *lnu*(A) and one *lnu*(B)], genes encoding the ABC-F proteins *vga* and *lsa* in one isolate each, and the *cfr* methylase gene in two isolates. One isolate had both *lnu*(B) and *lsa* (Table 3).

All MRSA had the *mecA* gene, and 80% (64/80) of MRSA and 29.8% (17/57) of MSSA had resistance genes to at least one of the three main aminoglycosides (gentamicin, tobramycin, or amikacin) (Supplementary Table S1). The predominant genes encoding resistance to aminoglycosides were the *aph (3*′*)-IIIa* gene encoding a phosphotransferase with resistance to amikacin (60/137, 43.8%), the *aadD1* gene encoding a nucleotidyl transferase with resistance to tobramycin (45/137, 32.8%), and the *aac* (6′)-Ie/*aph* (2″)-Ia encoding a two-domain acetyltransferase/phosphotransferase enzyme with resistance to the three aminoglycosides (12/137, 8.8%). The *aac* (6′)-Ie/*aph* (2″)-Ia gene was observed in 10% (8/80) of MRSA and 7% (4/57) of MSSA. No changes over time were observed for aminoglycoside resistance genes.

We identified mutations in the genes encoding topoisomerase II and/or topoisomerase IV in 53.3% (73/137) of the isolates, 78.7% (63/80) were in MRSA, and 17.5% (10/57) were in MSSA. Frequent mutations were S84L in *gyrA* (71/73, 97.3% of all isolates with mutations in this gene) and S80F in *parC* (95.9%) (Supplementary Table S1). Fifteen isolates had more than two mutations in topoisomerase II/IV genes.

### Phylogenetic analysis of erythromycin-resistant *Staphylococcus aureus* isolates by WGS

The 137 erythromycin-resistant *S. aureus* studied by MLST were grouped into 30 STs with an SDI of 21.9 and a mean of 4.6 isolates per ST (range = 1–45). The most prevalent STs (≥5 isolates) were ST125 (45, 32.8%), ST5 (16, 11.7%), ST398 (14, 10.2%), ST1 (11, 8%), ST30 (8, 5.8%), and ST8 (8, 5.8%), accounting for 74.4% of all STs (Supplementary Table S1).

**Figure 3:**
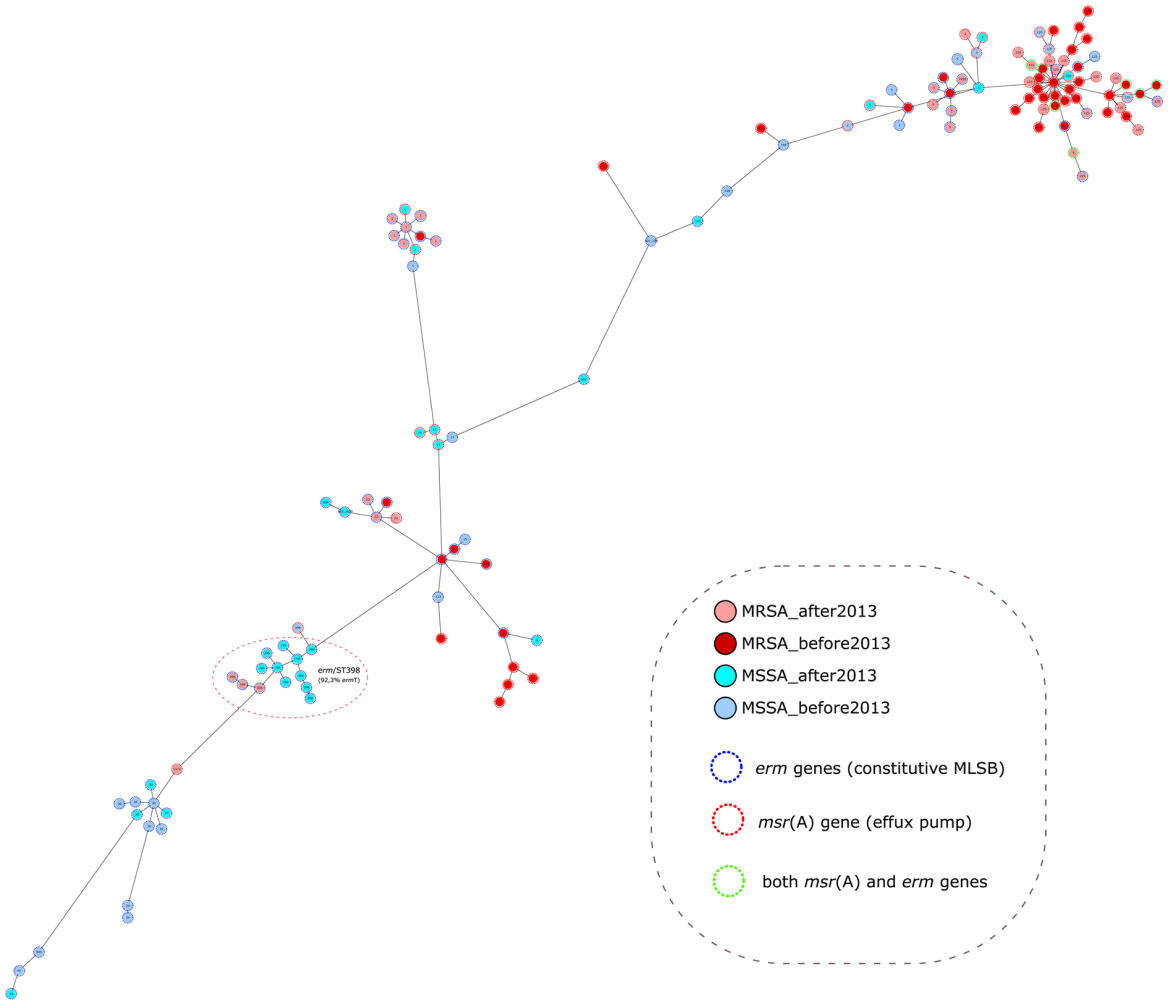
Population structure of *Staphylococcus aureus* isolates from this study: minimum-spanning tree. Distances shown are based on cgMLST of 1861 genes using the parameter “pairwise ignoring missing values.” Fill colors in each circle indicate MSSA and MRSA and the year of isolation, color of the dashed line in circles indicates macrolide resistance mechanism type.

Genome assemblies of 134 *S. aureus* isolates were analyzed using a gene-by-gene approach, and the allelic distance from cgMLST was visualized in a minimum spanning tree (Figure 3). All isolates differed by an average of 880 alleles (range = 0–1779), and the average allelic difference between ST125 isolates from this study was 60 (range = 0–121). ST5 isolates differed by an average of 188 alleles (range = 38–339); in ST398 isolates, it was 133 alleles (range = 1–265); in ST1 isolates, it was 126 alleles (range = 0–253); in ST30 isolates it was 213 alleles (range = 134–237); and in ST8 isolates, it was 155 alleles (range = 61–250).

ST125 was more prevalent in MRSA than in MSSA isolates (40/80 vs. 5/57, respectively, *p* < 0.0001); however, ST30 (0/80 vs. 8/57, respectively; *p* = 0.0007) and ST398 (3/80 vs. 11/57, respectively; *p* = 0.004) were more prevalent in MSSA (Figure 3). There were no differences in the distribution of STs in isolates before or after 2013, except for ST8, which was more prevalent in isolates prior to 2013 (7/74 vs. 1/63, *p* = 0.047), and ST398, which was more prevalent in isolates after 2013 (1/74 vs. 13/63, *p* = 0.0003).

Regarding to erythromycin-resistant mechanism, ST1, ST30 and ST398 were more frequent in *erm* isolates (10/82, 12.2%; 8/82, 9.6%; and 13/82, 15.8%; respectively) than in *msr*(A) isolates (1/63, 1.6%; 0/63; and 1/63, 1.6%; respectively) (*p* < 0.05). The prevalence of ST398/*erm*(T) in MSSA strains isolated after 2013 (10/29, 34.5%) was much higher compared to the other isolates (2/108, 1.8%) (*p* < 0.0001) (Figure 3).

**Figure 4:**
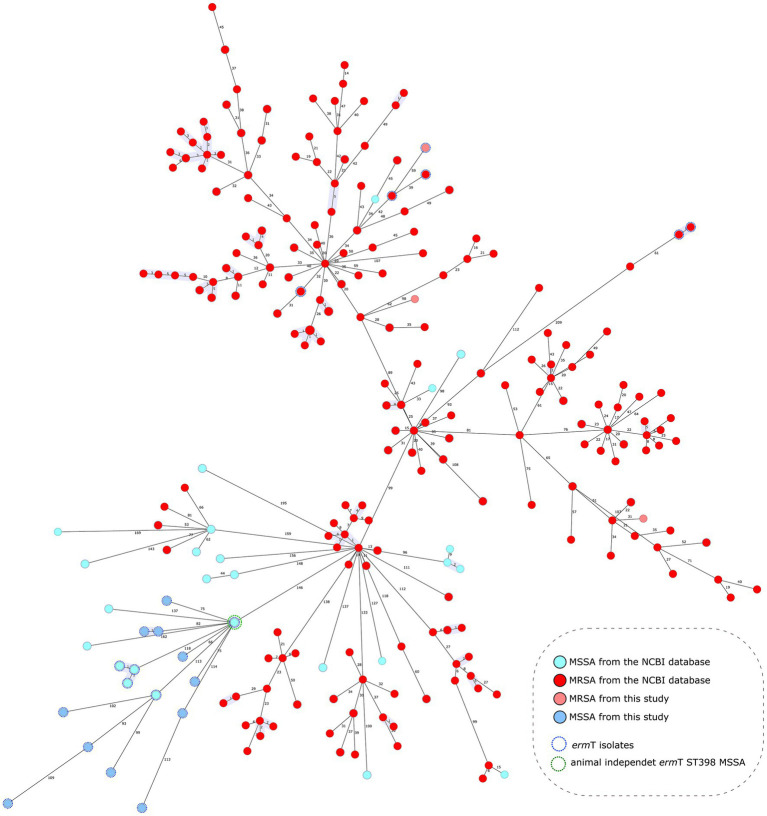
Population structure of ST398 *Staphylococcus aureus* in comparison with other publicly available whole genomes of the same ST. Minimum spanning tree, distance based in a cgMLST scheme of 1861 genes. Fill colors in each circle indicate MSSA and MRSA from this study or from other origins. Blue dashed line around circles remark isolates with *erm*(T) genes and green dashed line represent isolate NC_017631.1 (Uhlemann et al., 2012). Gray shadows represent groups of strains; a threshold of 5 alleles was applied.

The comparative analysis of ST398/*erm*(T) isolates included in this study with a collection *S. aureus* ST398 isolates from NCBI database showed that the most frequent mechanism of macrolide resistance was *erm* gene production, being *erm*(C) most frequent one followed by *erm*(A), *erm*(B) and *erm*(T). All MSSA isolates with *erm*(T) are grouped, MSSA *erm*(T) isolates from this study are grouped in a cluster together with animal-independent ST398 MSSA isolates reported in New York (Uhlemann et al., 2012) (Figure 4).

### Virulence genes in erythromycin-resistant *Staphylococcus aureus* isolates identified by WGS

The most common virulence genes (detected in ≥14 isolates) in the 137 isolates were the alpha-toxin gene *hla* (136, 99.3%), complement inhibitor gene *scn* (107, 78.1%), staphylokinase gene *sak* (90, 65.7%), chemotaxis inhibitory protein gene *chp* (87, 63.5%), beta-toxin gene *hlb* (32, 23.4%), hyaluronidase gene *hysA* (20, 14.6%), enterotoxin gene *sea* (15, 10.9%), and toxic shock syndrome toxin 1 gene *tsst*-1 (14, 10.2%) (Supplementary Table S1).

Other important virulence genes in *S. aureus*, such as Panton-Valentine leucocidin (PVL) genes *lukS* and *lukF*, and exfoliative toxin A gene *eta* were detected in eight (5.8%; 75% of them belonging to ST8) and six (4.4, 66.7% belonging to ST15) isolates, respectively (Supplementary Table S1). We found no pattern of virulence genes associated with the different groups, except for the presence of the *luk*F/*luK*S genes in the MRSA isolates. Of the genes mentioned above, the ST398/*erm*(T) isolates only presented homogeneously the *hla* (100%), *chp* (91.7%) and *scn* (91.7%) genes.

## Discussion

In addition to methicillin resistance, the cross-resistance to MLS_B_ antibiotics among *S. aureus* strains, as well as the rapid transmission of resistance genes, is a major concern for the future efficacy of antibiotic therapy. Specifically, the extensive use of MLS_B_ antibiotics against Gram-positive bacteria is of concern because macrolide-resistant MRSA strains are believed to be a major cause of clinical infections (Miklasińska-Majdanik, 2021), and they are associated with increased mortality rates (Bishr et al., 2021). In our study, erythromycin resistance in MRSA was approximately three times more common than in MSSA. However, our major concern that led to this research was the significant increase in erythromycin-resistant MSSA blood isolates that were not observed in MRSA.

The main strengths of this study were the analysis of bacteraemia caused by *S. aureus*, representing a broad national caseload over 17 years, in conjunction with antibiotic consumption data. Our molecular analyses represent a pilot study of a representative sample of *S. aureus* isolates to develop hypotheses about the EARS-Net findings, which will then require further studies. EARS-Net is coordinated by ECDC to collect, analyse, and report data on antimicrobial resistance through a network of national surveillance systems across Europe and to take actions to address antimicrobial resistance. During its more than 20 years of operation, EARS-Net has been effective in detecting changes in trends in antibiotic resistance in *S. aureus*, both at a national and a European level (Gagliotti et al., 2021).

The increase in erythromycin resistance in MSSA correlated temporally with an increase in the consumption of MLS_B_ in Spain; however, in 2016, outlier erythromycin resistance data reduced the statistical significance of the association. The cause of this single year of atypical data is unknown. The overall rise in MLS_B_ antibiotics consumption in this study was probably mostly due to the increased prescription of these antibiotics for community-acquired respiratory infections, and specifically the prescriptions of 3-day azithromycin courses. The association between macrolide consumption and increased resistance to MLS_B_ antibiotics has been described previously, especially in the context of specific pathologies such as cystic fibrosis (Tramper-Stranders et al., 2007) or trachoma (Bojang et al., 2017). Although the use of antibiotics (even appropriate usage) entails inevitable selection of resistance, this can be mitigated by the implementation of stewardship programs, including the diverse and combined use of antibiotics.

Although *erm*(C) is the most common macrolide resistance gene in *S. aureus* worldwide, it varies by geographical region and by the phenotype of susceptibility or resistance to methicillin (Miklasińska-Majdanik, 2021). Although we confirmed the general dominance of *erm*(C), we found a high prevalence of *msr*(A), which was most common in MRSA. A study in Spain (2006–2007) showed 23.1% prevalence for *erm* genes and 15.8% for *msr* genes in invasive *S. aureus* (Pérez-Vázquez et al., 2009).

The present study showed a strong association between *msr*(A) and *mph*(C) genes; only 2/63 isolates with *msr*(A) lacked *mph*(C). Matsuoka et al. (2003) found the *mph*(C) phosphotransferase gene on plasmid pMS97, 342 bp downstream of the *msr*(A) gene. These authors suggested, based on their findings, that a region of the *msr*(A) gene is required for the full expression of *mph*(C). However, that plasmid also carried *erm*(Y) (15), a gene that we did not find in our study.

One of the main findings of this study was the prevalence of *erm* (T)-bearing ST398 isolates in MSSA collected during the period in which EARS-Net found a significant increase in erythromycin resistance in MSSA isolates that produced bacteraemia. Although this pilot study included a limited number of representative isolates, our finding suggests that the increased resistance to macrolides in these MSSA identified by EARS-Net could be due, at least in part, to the spread of ST398 in this group of isolates. ST398 is relevant to public health because methicillin-resistant strains are associated with livestock capable of infecting humans (Silva et al., 2023). However, previous studies suggested two subpopulations in clonal complex 398 *S. aureus*—a human-adapted clade mostly with the *erm*(T) gene, and an animal-associated clade with *mecA*, *tet*(M), and *erm*(C) genes (Price et al., 2012; Argudín et al., 2018). A recent Belgian study identified different ST398 subpopulations, including typical human and animal clades, as well as new emerging mixed subpopulations that underlie the ability of this lineage to acquire resistance and virulence genes (Argudín et al., 2018). The proximity of *erm*(T) and *rep*13 genes in MSSA ST398 isolates suggests that *erm*(T) is on a plasmid, as has been described recently (Salgueiro et al., 2023). The MSSA ST398 human clade was reported mainly in China and France (Bouiller et al., 2020), and was frequently implicated in severe infections, whereas the MRSA ST398 animal clade was reported mainly in the skin and soft tissue (Bouiller et al., 2020).

Most well-known staphylococcal virulence genes, such as enterotoxins, toxic shock syndrome toxin, or PVL, were absent in ST398 isolates from this study, as previously communicated (Bouiller et al., 2020). However, previous studies have suggested that MSSA ST398, which is prevalent in bacteraemia, may be a more virulent subtype (Bouiller et al., 2020) with higher mortality (Bouiller et al., 2016). Among the virulence factors previously associated with ST398 MSSA, only *scn* and *chp* genes were identified in *erm*(T) ST398 isolates of our study, but the immune evasion cluster (IEC) *sak* and *sea* genes were not found.

A limitation of this research could be the reduced number of sequenced isolates by group, which, although they were strictly chosen to be representative of the issue to study, not all belonged to the same collection in which the increase in resistance was primarily detected.

## Conclusion

The highly significant increase in resistance to macrolides in MSSA causing bacteraemia, in contrast to the absence of this trend in MRSA, correlated with the consumption of MLS_B_ antibiotics in Spain. Our data seem to support the hypothesis that the human ST398 MSSA clade with *erm*(T)-mediated resistance to erythromycin may be involved in this trend. An epidemiological surveillance system for MRSA and MSSA is important to monitor the emergence of dangerous new *S. aureus* subpopulations. Further WGS research is needed to identify the emergence of these subpopulations in the clinical setting, as well as their correlation with changes in the patterns of antibiotic susceptibility.

## Data availability statement

The data presented in the study are deposited in the European Nucleotide Archive (ENA) repository (https://www.ebi.ac.uk/ena), accession number PRJEB61102.

## Ethics statement

Ethical review and approval was not required for the study on human participants in accordance with the local legislation and institutional requirements. Written informed consent for participation was not required for this study in accordance with the national legislation and the institutional requirements.

## Spanish EARS-Net group

Members of the Spanish EARS-Net group are as follows: Amparo Coira Nieto and Daniel Navarro de la Cruz (EOXI de Santiago de Compostela, A Coruña); Elena Escribano, Mª Dolores Crespo and Juan José Palomar (H. Universitario de Albacete, Albacete); Mónica Parra (H. General Universitario de Alicante, Alicante); Nieves Gonzalo and Gloria Royo (H. General de Elche, Elche, Alicante); María Navarro and Nieves Gonzalo (H. de Orihuela, Orihuela, Alicante); Teresa Cabezas (H. de Poniente, El Ejido, Almería); Javier Fernández Domínguez and Ana Mª Fleites (H. Universitario Central de Asturias, Oviedo, Asturias); Eugenio Garduño (H. Infanta Cristina, Badajoz); Belén Viñado and Oscar del Valle (H. Universitari Vall d’Hebrón, Barcelona); Isabel Sanfeliú and Dionisia Fontanals (Corporació Sanitaria Parc Taulí, Sabadell, Barcelona); José Lite and Javier Garau (H. Mutua Terrassa, Terrassa, Barcelona); Pilar Berdonces (H. de Galdakao, Bizkaia); Mª Angeles Mantecón and Eva Ojeda (Complejo Asistencial de Burgos, Burgos); Rosario Sánchez Benito, Jesús Viñuelas and Pilar Teno (H. San Pedro de Alcántara, Cáceres); Carmen Martínez Rubio, Carolina Freyre and Manuel Antonio Rodríguez Iglesias (H. Universitario de Puerto Real, Cádiz); Natalia Montiel and Manuel Antonio Rodríguez Iglesias (H. Universitario Puerta del Mar, Cádiz); José Luis de Francisco Ramírez, Juan Carlos Alados and Mª Dolores López Prieto (H. Universitario de Jerez de la Frontera, Cádiz); Susana Sabater and Rosario Moreno (H. General de Castellón, Castellón); Alberto Yagüe, Óscar Pérez Olaso and María Gil Fortuño (H. La Plana, Villareal, Castellón); Ana Bordes Benítez and Raúl Guilarranz Luengo (H. Universitario Dr. Negrín, Las Palmas de Gran Canaria); Fernando Cobo Martínez, Mª Dolores Pérez Ramírez and Dolores Rojo (H. Virgen de las Nieves, Granada); Alejandro González Praetorius (H. Universitario de Guadalajara, Guadalajara); José Antonio Lepe (H. Río tinto, Huelva); Inocente Cuesta and Concepción Carazo (H. Universitario Ciudad de Jaén, Jáen); Carmen Amores (H. San Agustín, Linares, Jaén); M. José Gastañares (H. San Millán, Logroño, La Rioja); Isabel Fernández- Natal (Complejo Asistencial Universitario de León, León); María Rodríguez Velasco, Carmen Raya and Carlos Fúster (H. del Bierzo, Ponferrada, León); Ana Mª Sánchez Díaz, Elena Loza and Ana Verónica Halperin (H. Universitario Ramón y Cajal, Madrid); Mª José González and Mercedes Menéndez Rivas (H. Niño Jesús, Madrid); José Francisco Valverde Cánovas and Alberto Delgado Iribarren (Fundación H. de Alcorcón, Madrid); Jesús García Martínez (H. Universitario de Fuenlabrada, Madrid); Sara Quevedo, Isabel Wilhelmi and Pilar Reyes (H. Severo Ochoa, Leganés, Madrid); Isabel Sánchez Romero (H. General Universitario Puerta de Hierro, Majadahonda, Madrid); Mª Victoria García-López and Alfonso Pinedo (H. Virgen de la Victoria, Málaga); Fernando Fernández Sánchez and Natalia Montiel (H. Costa del Sol, Marbella, Málaga); Xabier Berastain (H. Universitario de Navarra, Navarra); Isabel Paz Vidal, Begoña Fernández, Gloria Esteban and Almudena Tinajas (Complejo Hospìtalario de Orense, Orense); Almudena Tinajas (Complejo Hospitalario Asistencial de Palencia); Antonio Oliver and José Luis Pérez (H universitario son Espases, Palma de Mallorca); Mª Ángeles Pallarés, Marta García Campello, Victoria Pullian and Mª Ángeles Pascual (Complejo Hospitalario de Pontevedra, Pontevedra); Francisco Vasallo (H. Universitario de Vigo, Pontevedra); Ana Isabel Aller, Estrella Martín and Samuel Bernal (H. Universitario Valme, Sevilla); José Antonio Lepe (H. Universitario Virgen del Rocío, Sevilla); Carmen Aldea Mansilla, Ángel Campos and Teresa Nebreda (Complejo Asistencial de Soria, Soria); Mar Olga Pérez Moreno, Ignacio Buj and Mª José Centelles (H. Virgen de la Cinta, Tortosa, Tarragona); Teresa Delgado Melián, Mª Antonia de Miguel and Antonio Sierra (H. Universitario de Canarias, Tenerife); Juan Frasquet (H. Universitari i Politècnic La Fe, Valencia); Mª Fe Brezmes and Juana Rodríguez-Hernández (H. Virgen de la Concha, Zamora); Ana López Caleja, Mª Cruz Villuendas, Javier Pereira, Luisa Marco and Mª José Revillo (H. Universitario Miguel Servet, Zaragoza); Carmen Aspiroz (H. Royo Villanova, Zaragoza).

## Author contributions

JO-I, BA, and MP-V conceived, designed, and coordinated the study. AE, ERa, JC-G, MP-V, VC-G, JL, ERu, JC-M, NL, RC, EC, SG-C, and Spanish EARS-Net Group collected data and isolates and performed the experiments. AE, BA, and JO-I analyzed resistance trends. LV-G and JO-I collected and analyzed antibiotic consumption trends. JO-I, AE, ERa, JC-G, and MP-V wrote the manuscript. All authors have read, edited and approved the final manuscript.

## Footnotes

^1^ https://www.eucast.org/clinical_breakpoints

^2^http://quast.bioinf.spbau.ru/, accessed on 3 March 2023.

^3^ https://pubmlst.org/organisms/staphylococcus-aureus

^4^https://card.mcmaster.ca, accessed on 3 December 2022.

^5^https://cge.cbs.dtu.dk, accessed on 3 December 2022.

^6^https://bitbucket.org/genomicepidemiology/virulencefinder_db/src/master/, version 2022-12-02.
